# The Predictive Value of Waist-To-Height Ratio for Ischemic Stroke in a Population-Based Prospective Cohort Study among Mongolian Men in China

**DOI:** 10.1371/journal.pone.0110245

**Published:** 2014-10-29

**Authors:** Juan Xu, Tian Xu, Xiaoqing Bu, Hao Peng, Hongmei Li, Mingzhi Zhang, Yonghong Zhang

**Affiliations:** Department of Epidemiology, School of Public Health, Medical College of Soochow University, Suzhou, China; National Cancer Center, Japan

## Abstract

**Objective:**

To explore the associations between waist-to-height ratio (WHtR), body mass index (BMI) and waist circumference (WC) and risk of ischemic stroke among Mongolian men in China.

**Methods:**

A population-based prospective cohort study was conducted from June 2003 to July 2012 in Inner Mongolia, an autonomous region in north China. A total of 1034 men aged 20 years and older free of cardiovascular disease were included in the cohort and followed up for an average of 9.2 years. The subjects were divided into four groups by WHtR levels (WHtR<0.40, 0.40≤WHtR≤0.50, 0.50<WHtR≤0.60, WHtR>0.60). The cumulative survival rates of ischemic stroke among the four groups were estimated with the Kaplan-Meier curves and compared by log-rank test. Cox proportional hazards models and Receiver Operating Characteristic (ROC) curves were employed to evaluate the associations between obesity indices and ischemic stroke.

**Results:**

A total of 47 ischemic stroke patients were observed during the follow-up period. The cumulative incidence and incidence density of ischemic stroke were 4.55% and 507.61/100 000 person-years, respectively. After the major risk factors were adjusted, individuals with WHtR>0.60 had a 3.56-fold increased risk of ischemic stroke compared with those with 0.40≤WHtR≤0.50. Hazard ratio (HR) and 95% confidence intervals (CI) of ischemic stroke for a 1-SD increase in WHtR was 1.34(95% CI: 1.00–1.81). After adding BMI or WC to models, higher WHtR remained significantly associated with increased risk of ischemic stroke. The Kaplan-Meier survival curves showed that the cumulative survival rate in the group with WHtR>0.60 was significantly lower than in the group with 0.40≤WHtR≤0.50 (log-rank test, *P* = 0.025). The areas under the curve for each index were as follows: 0.586 for WHtR, 0.543 for WC; 0.566 for BMI.

**Conclusions:**

Higher WHtR is associated with risk of ischemic stroke in Mongolian males. WHtR may be useful in predicting ischemic stroke incidence in males.

## Introduction

Stroke is the second cause of death and leading cause of long-term disability worldwide [1], [2]. It is now becoming the first leading cause of death in China [3]. Ischemic stroke is the most common kind of stroke and accounts for 43%–79% of all stroke cases [1], [4]. The prevalence of obesity and obesity related chronic diseases (e.g. hypertension, stroke, and diabetes mellitus) have increased during the past years in China [5], [6]. Body mass index (BMI) is the most common anthropometric index for assessing body fat and diagnosing overweight and obesity in clinical practice and epidemiologic research. Waist circumference (WC), as an indicator of abdominal adiposity, is a predictive factor of cardiovascular disease (CVD) [7]. However, the relative practicability of BMI or WC has been questioned and the relationships between these obesity indices and cardiovascular risk are still controversial [7], [8]. Some epidemiological studies have showed that waist-to-height ratio(WHtR) correlates better than BMI and WC with hypertension, diabetes mellitus, and cardiovascular diseases [9]–[11]. Our previous study showed there were significant differences in most risk factors of stroke between Mongolian men and women at the baseline and the incidence rate of ischemic stroke for males was higher than for females [12], [13]. Thus, the purpose of this study was to examine the associations between adiposity indices (WHtR, BMI and WC) and ischemic stroke among Mongolian men.

## Materials and Methods

### Study participants

The baseline data was collected from May 2002 to June 2003 in Inner Mongolia, an autonomous region in north China. A cluster sampling method was adopted in the study. Two townships including 32 villages in Kezuohou Banner and Naiman Banner in Inner Mongolia were selected as the investigation fields. The majority of local residents were Mongolians who had lived there for many generations, their professions were farmers and herdsmen and they maintained a traditional diet that was high in fat and salt. A total of 3475 (1677 men and 1798 women) Mongolian people were eligible in the 32 villages. Among them, 2589 (1064 men and 1524 women) individuals completed the baseline survey. The recruitment rate was 74.50%. In this study, we examined the associations between adiposity indices (WHtR, BMI and WC) and ischemic stroke among Mongolian men. This study was approved by the Soochow University Ethics Committee in China. Written informed consent was obtained for all study participants.

### Data Collection

All the participants took part in the face-to-face interviews and physical examinations in the baseline survey. Information on demographic characteristics, personal medical history, and lifestyle risk factors was obtained using a standard questionnaire administered by trained staff. Cigarette smoking was defined as having smoked at least one cigarette per day for 1 year or more. Alcohol consumption was defined as consuming at least 50 g distillate spirits per day for 1 year or more. Three consecutive sitting blood pressure measurements were taken for each participant by trained staff using a mercury sphygmomanometer according to a standard protocol, after the subjects had been resting for at least 30 minutes. The first and fifth Korotkoff sounds were recorded as systolic blood pressure (SBP) and diastolic blood pressure (DBP), respectively. The mean of the three records was used in analysis. Standing height was measured with a fixed stadiometer calibrated in centimeters and body weight was measured in kilograms by using a balance-beam scale with participants wearing light clothing and no shoes. BMI was calculated as the ratio of weight in kilograms to height in meters squared. Base on the recommendations of the Working Group on Obesity in China, participants were stratified according to the BMI levels as underweight group (BMI<18.5 kg/m^2^), normal weight group (18.5 kg/m^2^≤BMI<24.0 kg/m^2^), overweight (24.0 kg/m^2^≤BMI<28.0 kg/m^2^), or obesity (BMI≥28.0 kg/m^2^). WC was measured 1 cm above the umbilicus. Abdominal obesity was defined as WC≥85 cm for male [14]. WHtR was calculated as WC divided by height. According to the reports from Ashwell, we categorized WHtR as WHtR<0.40, 0.40≤WHtR≤0.50, 0.50<WHtR≤0.60, WHtR>0.60. The group with 0.40≤WHtR≤0.50 was used as the reference group [15], [16].

All blood samples were obtained from the antecubital vein in the morning after a requested overnight fast (at least 8 hours); all plasma and serum samples were frozen at −80°C until testing. Fasting plasma glucose (FPG) was measured using a modified hexokinase enzymatic method. Total cholesterol (TC), high density lipoprotein cholesterol (HDL-C), and triglyceride (TG) were analyzed enzymatically using a Beckman Synchron CX5 Delta Clinical System (Beckman Coulter, Inc; Fullerton, CA) with commercial reagents. Low density lipoprotein cholesterol (LDL-C) concentration was calculated by use of the Friedewald equation for the participants who had TG levels <400 mg/dL [17].

### Follow-up and Outcome Assessment

All study participants were followed from June 2003 through July 2012. Ischemic stroke events during follow-up were the primary study outcome. Participants who did not have an ischemic stroke, who died from other causes, or who were lost to follow-up were defined as censored. If the participant was interviewed and found to have had an ischemic stroke, the ischemic stroke incidence date was defined as the end point date. Data were censored at the time of the contact if the participant was reached and found not to have an ischemic stroke. Data were censored at the time of death date in the medical record for those who died from other causes. Data were censored at the day we contacted the participant last time if he was lost to follow-up. Since 2004, household surveys of the participants were conducted every 2 years to determine new ischemic stroke cases. Trained staff interviewed either the participants or their relatives, if participants were dead or unable to communicate, and completed a medical status questionnaire. At last, if the subjects reported that an ischemic stroke occurred during the period since the last survey, the staff reviewed hospital records, including outpatient or admission records, the discharge summary. Only the subjects who were diagnosed with ischemic stroke by head computed tomography or MRI scan at the hospital were considered to have the outcome of interest in this study.

### Statistical Analysis

The participants were initially categorized into four groups based on WHtR levels (WHtR<0.40, 0.40≤WHtR≤0.50, 0.50<WHtR≤0.60, WHtR>0.60). Data of baseline characteristics were expressed as means (standard deviations), medians (quartile intervals range), or n (%) for the four groups, respectively. The four groups were compared using analysis of variance (ANOVA) or Kruskal-Wallis rank test. Student-Newman-Keuls (SNK) or Nemenyi rank tests were used for comparison between each two groups. Proportions for categorical variables were calculated and χ^2^ tests were performed to compare between/among groups. Person-years of follow-up were calculated as the time from baseline assessment to development of the first endpoint of interest, censoring, or the end of follow-up, whichever occurred first. Univariate and multivariate Cox proportional hazards models were used to assess the associations between WHtR, BMI, WC and the risk of ischemic stroke. Hazard ratios (HRs) and 95% confidence intervals (CIs) of ischemic stroke were calculated for abnormal groups (WHtR<0.40, 0.50<WHtR≤0.60, WHtR>0.60) with the normal group (0.40≤WHtR≤0.50). The HRs (95%CI) of ischemic stroke across increasing WHtR, BMI, and WC levels were determined. Cumulative survival rates for the four groups with different values of WHtR were estimated by Kaplan-Meier survival curves, and probability values (*P*-values) were determined with the log-rank test. We assessed the discriminatory value of the three anthropometric indices for predicting ischemic stroke by computing Receiver Operating Characteristic (ROC) curves and comparing the areas under ROC curves (AUCs) with the Z-statistic (MedCalc 5.0, MedCalc Inc). All reported *P*-values were 2-tailed, and a significance level of 0.05 was used. Statistical analysis was conducted using SAS statistical software (version 9.1, Cary, North Carolona, USA).

## Results

As of July 31, 2012, we have followed participants for an average of 9.2 years. Among 1064 male participants, 3 were lost to follow-up, and the follow-up rate was 99.72%. After exclusion of 30 individuals for lacking the blood samples or anthropometric indices, a total of 1034 people were included in the final analysis. A total of 47 patients with ischemic stroke were present during the follow-up period. The cumulative incidence rate of ischemic stroke was 4.55% and the incidence density was 507.61 per 100 000 person-years. A total of 75 people died from other causes in this cohort study among 1034 participants. Baseline characteristics of participants stratified according to the values of WHtR were presented in Table 1. Conventional stroke risk factors such as age, smoking, alcohol consumption, FPG, TC, TG, HDL-C, LDL-C, BMI, WC, SBP and DBP were significantly different among the four groups. Compared with the reference group (0.40≤WHtR≤0.50), the participants with 0.50<WHtR≤0.60 tended to be older, and had higher FPG, TC, TG, LDL-C, BMI, WC, SBP, DBP and lower HDL-C. Participants with WHtR>0.60 tended to be smoker, and had higher FPG, SBP, DBP, TC, TG, LDL-C, BMI, WC and lower HDL-C. Participants with WHtR<0.40 had lower BMI and WC. No significant differences in the rates of family history of cardiovascular disease were found among the four groups.

**Table 1:** Baseline Characteristics of Participants Stratified According to Values of WHtR.

	WHtR<0.40	0.40≤WHtR≤0.50,	0.50<WHtR≤0.60	WHtR>0.60	*P*
n	20	668	308	38	-
Age, years	44.55±11.55	46.13±12.87	50.25±12.29*	49.95±12.23	<0.001
Smoking, %	16 (80.00)	446 (66.77)	187 (60.71)	15 (39.47)*	<0.001
Alcohol consumption, %	9 (45.00)	428 (64.07)	209 (67.86)	24 (63.16)	0.003
Family history of CVD, %	5 (25.00)	125 (18.71)	77 (25.00)	12 (31.58)	0.199
Systolic blood pressure, mmHg	128.40±32.39	129.20±22.30	137.46±24.43*	147.79±26.58*	<0.001
Diastolic blood pressure, mmHg	85.15±12.42	85.03±11.84	90.68±12.17*	97.34±15.94*	<0.001
Fasting plasma glucose, mmol/L	4.70 (4.20 5.05)	4.70 (4.20 5.30)	5.10 (4.60 5.70)*	5.30 (4.90 5.80)*	<0.001
total cholesterol, mmol/L	3.12 (2.64 3.85)	3.45 (2.85 4.10)	3.97 (3.25 4.86)*	4.53 (3.59 5.58)*	<0.001
triglyceride, mmol/L	0.59 (0.51 0.98)	0.94 (0.68 1.39)	1.28 (0.87 2.28)*	2.00 (1.11 3.33)*	<0.001
HDL-C, mmol/L	1.19 (1.08 1.40)	1.17 (0.97 1.39)	1.08 (0.91 1.25)*	0.97 (0.81 1.08)*	<0.001
LDL-C, mmol/L	1.66 (1.35 2.29)	2.00 (1.52 2.58)	2.47 (1.82 3.25)*	3.07 (2.26 3.83)*	<0.001
Body mass index, kg/m^2^	18.14 (17.6319.21)*	20.35 (19.20 21.63)	24.20 (22.78 26.00)*	29.13 (27.90 31.56)*	<0.001
Waist circumference, cm	68 (66 70)*	77 (74 80)	90 (86 94)*	106(103 110)*	<0.001

WHtR, waist-to-height ratio; CVD, cardiovascular disease; HDL-C, high density lipoprotein cholesterol; LDL-C, low density lipoprotein cholesterol.

*Compared with 0.40≤WHtR≤0.50, *P*<0.05.

Table 2 summarizes the HRs for ischemic stroke according to the levels of three adiposity indices. Univariate analysis showed that the HR of ischemic stroke for participants with WHtR>0.60 increased by 2.17-fold (*P* = 0.033), compared with the reference group. After adjustment for age, smoking, alcohol consumption, family history of cardiovascular disease, FPG, TC, TG, HDL-C, and DBP, risk of ischemic stroke for participants with WHtR>0.60 increased 256% compared with the reference group (HR = 3.56, *P* = 0.033). HRs of ischemic stroke for the high WC (≥85 cm) were not significant compared to those with normal WC (<85 cm) in either univariate or multivariate models. HRs of ischemic stroke for three groups of BMI (underweight, overweight, and obesity) were not significant compared to normal weight group in either univariate or multivariate models.

**Table 2:** Hazard Ratios and 95% Confidence Intervals of Ischemic Stroke associated with different Values of WHtR, BMI and WC.

		Un-adjusted		Adjusted*	
	cases	HR (95% CI)	*P-* value	HR (95% CI)	*P-* value
WHtR					
<0.40	2	2.83 (0.67–11.98)	0.157	2.76 (0.62–12.31)	0.182
0.40–0.50	24	1	-	1	-
0.51–0.60	17	1.53 (0.82–2.85)	0.178	1.09 (0.57–2.07)	0.795
>0.60	4	3.17 (1.10–9.15)	0.033	3.56 (1.11–11.44)	0.033
BMI, kg/m^2^					
<18.5	8	2.15 (0.98–4.70)	0.056	1.28 (0.57–2.88)	0.552
18.5–23.9	29	1	-	1	-
24–27.9	7	0.98 (0.43–2.23)	0.952	1.12 (0.48–2.63)	0.793
≥28	3	1.42 (0.43–4.67)	0.562	2.90 (0.78–10.87)	0.114
WC, cm					
<85	27	1	-	1	-
≥85	20	1.59 (0.89–2.84)	0.115	1.40 (0.76–2.58)	0.287

WHtR, waist-to-height ratio; BMI, body mass index; WC, waist circumference.

*Adjust for age, smoking, alcohol consumption, family history of cardiovascular disease, diastolic blood pressure, fasting plasma glucose, high density lipoprotein cholesterol, total cholesterol, and triglyceride.

As shown in Table 3, the multivariate-adjusted HR (95% CI) for each 1-SD in WHtR was 1.34 (95% CI: 1.00–1.81) for ischemic stroke. After adding BMI or WC to models, higher WHtR remained a significant association with increased risk of ischemic stroke.

**Table 3:** Hazard Ratios and 95% Confidence Intervals of Ischemic Stroke for a 1-SD Increase in Anthropometric Indicator.

	Un-adjusted		Adjusted*	
	HR (95% CI)	*P-*value	HR (95% CI)	*P-*value
WHtR				
Increase per SD	1.34 (1.02−1.75)	0.035	1.34 (1.00−1.81)	0.053
Adding adjustment for BMI			2.26 (1.71−2.98)#	<0.001
Adding adjustment for WC			5.16 (2.22−12.03)	<0.001
BMI				
Increase per SD	0.81 (0.59−1.12)	0.211	0.78 (0.56−1.09)	0.141
WC				
Increase per SD	1.14 (0.87−1.50)	0.338	1.01 (0.82−1.48)	0.522

WHtR, waist-to-height ratio; BMI, body mass index; WC, waist circumference.

*adjustment for smoking, alcohol consumption, family history of cardiovascular disease, diastolic blood pressure, fasting plasma glucose, high density lipoprotein cholesterol, total cholesterol, and triglyceride;

# adding adjustment for BMI;

adding adjustment for WC.

**Figure 1:**
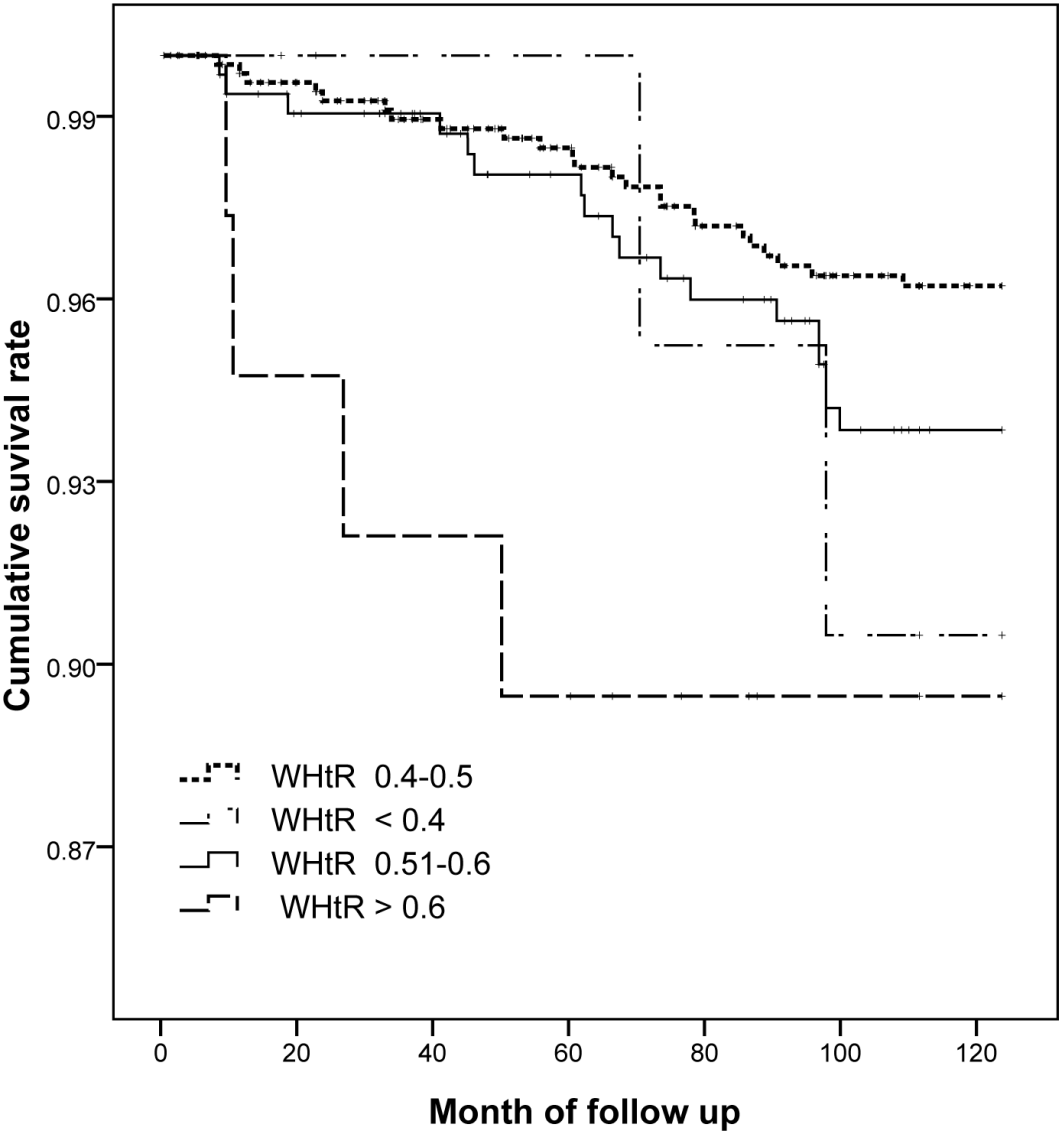
Kaplan-Meier curves of participants for ischemic stroke according the values of WHtR at baseline. WHtR, waist-to-height ratio.

**Figure 2:**
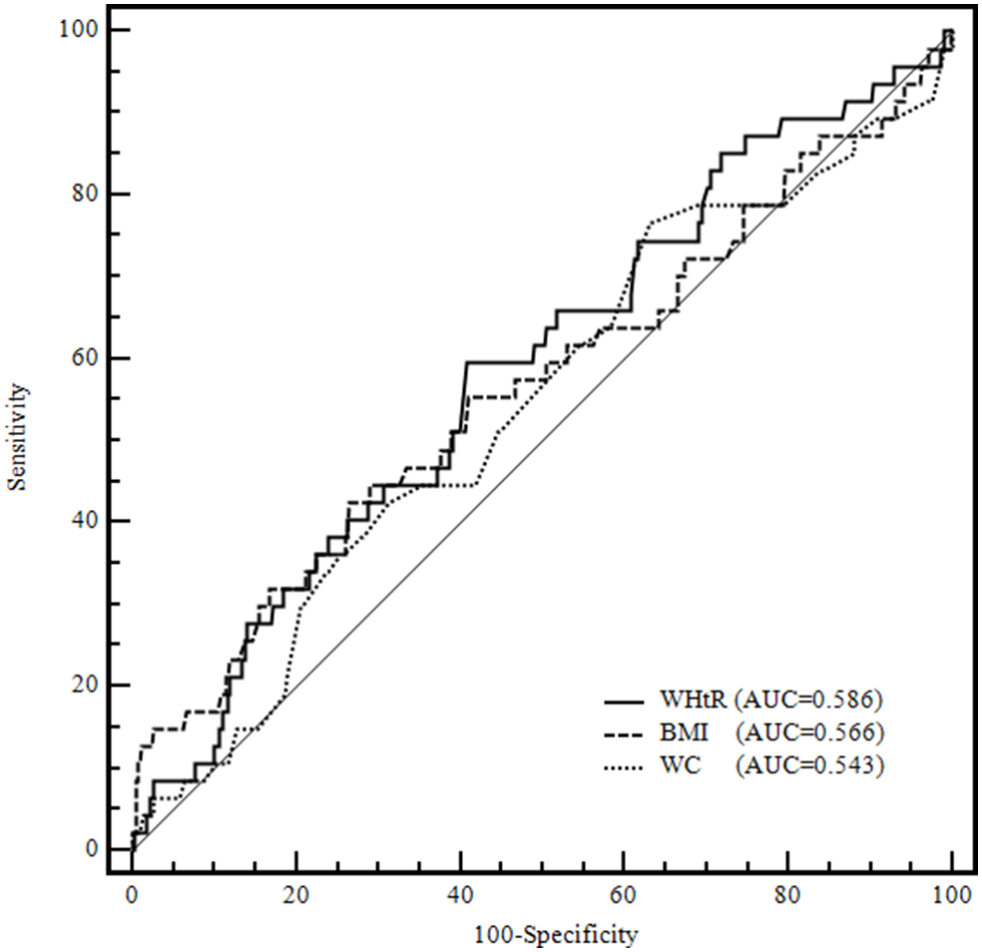
Prediction of ischemic stroke by receiver operating characteristic (ROC) curves for WHtR, BMI and WC. WHtR, waist-to-height ratio; BMI, body mass index; WC, waist circumference.

The Kaplan-Meier survival curves for the four groups according to the values of WHtR were showed in Figure 1. The curves showed that the cumulative survival rate in group with WHtR>0.60 was lowest among four groups and was significantly lower than that in the group with 0.40≤WHtR≤0.50 (log-rank test, *P* = 0.025). Figure 2 showed ROC curves for three anthropometric indices (BMI, WC and WHtR). The AUC values for each index were as follows: 0.543 for WC; 0.566 for BMI; 0.586 for WHtR. WHtR was significantly better than WC in predicting ischemic stroke, with a significance level of *P* = 0.005. The AUC of WHtR was larger than BMI although they were not significantly different (*P* = 0.797). We did not find a significant difference in the AUC values between BMI and WC (*P* = 0.818).

## Discussion

In this prospective cohort study, WHtR was associated with risk of ischemic stroke in Mongolian males. Individuals with WHtR>0.60 had a 3.56-fold increased risk of ischemic stroke compared with those with 0.40≤WHtR≤0.50, after the major risk factors were adjusted.

There is a geographical and ethnic difference of stroke incidence in China [18]. Mongolian is a minority living in the north of China and maintains the custom of Mongolian ethnicity, which differs from Chinese Han people. An epidemiological investigation among 9146 Mongolian and Han residents in pastoral area of Inner Mongolia showed that Mongolian residents had higher levels of WC (87.27 vs 86.01 cm), BMI (24.59 vs 24.25 kg/m^2^) and WHtR (0.55 vs 0.54) than Han residents (all *P*<0.01) [19].The incidence rate of stroke was much higher in Mongolian population than the national average level in China [20], [21]. Therefore, our study indicates that WHtR may be a useful obesity index in predicting ischemic stroke incidence among Mongolian men.

The significant association of WHtR with risk of ischemic stroke in our study is consistent with the findings in earlier studies among Han people [22], [23]. The 6.3-year cohort study among Han people in Anqing rural areas of China showed that WHtR was associated with ischemic stroke in men, but no significant association was observed after additionally adjusted for SBP and DBP [23]. Bodenant et al [8] found that WHtR was associated more strongly with stroke than BMI in men. After adjustment for BMI, the relative risk for stroke associated with WHtR remained significant. Furthermore, WHtR was also associated with the risk of stroke in men with normal weight and less than normal weight, but the association of WC with stroke was no significant in these men. Another recent report showed extreme WHtR values could independently predict 12-month all-cause mortality in patients with acute ischemic stroke but BMI was not [24]. The results suggested that WHtR may improve the assessment of stroke risk in people whose risk may be underestimated by BMI.

BMI and WC, as the current standard measure of adiposity, may have several limitations. A general limitation of BMI is that it does not distinguish the subjects with excess adipose tissue from those with heavy muscle mass. WC is an indicator of abdominal fat content, but the normal range of WC is various in different populations. It may incorrectly evaluate risk of stroke for populations with different height. WHtR, as a simple, rapid screening tool, can help to overcome the limitations in the use of BMI or WC. It is a pragmatic indicator of obesity that accurately demonstrates body fat excess in the population with different race, age and gender. Recently, a meta-analysis demonstrated that WHtR was superior to BMI and WC for predicting CVD risk [10]. A study by Gelber et al showed that WHtR had the strongest association with CVD in 1 6332 men [25]. Moreover, the superiority of WHtR for detecting CVD risk has been reported among children and adolescents [26]. Therefore, WHtR may be considered as a good screening tool for CVD including ischemic stroke.

Some studies have shown that obesity was related to endothelial dysfunction, inflammatory reaction and tissue injury responses [27], [28]. A study reported that WHtR was a more sensitive obesity index associated with chronic inflammation [29]. The potential mechanism linking WHtR to an increased the risk of stroke is that high WHtR may increase chronic inflammation reaction.

This study has several strengths that deserve mentioning. To our knowledge, it is the first study to examine the association between WHtR and ischemic stroke in Mongolian males. In addition, the participants were homogeneous regarding their genetic background, and living conditions as well as lifestyles. The study data were collected with rigid quality control, and important co-variables were measured and controlled in the analysis. Furthermore, our follow-up time is relatively long, which enable us to get a less biased association between exposure variables and outcome events. However, the results of ROC analysis suggested that anthropometric indexes were not good discriminate predictors for ischemic stroke in this study. Therefore, some additional factors besides anthropometric indexes should be considered in preventing ischemic stroke, such as controls of blood pressure, smoking or drinking behavior [12], [30].

There were some limitations in this study. First, the study only recognized association between WHtR and ischemic stroke in Mongolian men, and might not be representative of the other Chinese populations with different races. Second, the anthropometric markers were measured only once at baseline, therefore intraindividual variation during the follow-up could not be assessed. In addition, approximately 25% of the eligible population from the 32 villages did not participate, which may have introduced some selection bias. We suggest that this bias is minimal because it is unlikely that participants chose not to participate due to their anthropometric indexes. Finally, although the important effect was observed in the highest WHtR category, the number of ischemic stroke events might be relatively few and larger-sample cohort studies are still required to examine these associations between obesity indices and ischemic stroke.

## Conclusions

In summary, our study reveals that in Mongolian males, higher WHtR is associated with risk of ischemic stroke. WHtR may be useful in predicting ischemic stroke incidence in males.
